# Study of siRNA Delivery via Polymeric Nanoparticles in Combination with Angiogenesis Inhibitor for The Treatment of *AFP*-Related Liver Cancer

**DOI:** 10.3390/ijms232012666

**Published:** 2022-10-21

**Authors:** Kittiporn Punuch, Chamaiphorn Wongwan, Saranrat Jantana, Chayapol Somboonyosdech, Kamonlatth Rodponthukwaji, Natsuda Kunwong, Kytai T. Nguyen, Vorapan Sirivatanauksorn, Yongyut Sirivatanauksorn, Chatchawan Srisawat, Primana Punnakitikashem

**Affiliations:** 1 Department of Medicine, Faculty of Medicine Siriraj Hospital, Mahidol University, Bangkok 10700, Thailand; 2 Department of Biochemistry, Faculty of Medicine Siriraj Hospital, Mahidol University, Bangkok 10700, Thailand; 3 Research Network NANOTEC—MU in Theranostic Nanomedicine, Bangkok 10700, Thailand; 4 Department of Bioengineering, University of Texas at Arlington, Arlington, TX 76019, USA; 5 Siriraj Center of Research Excellence in Theranostic Nanomedicine, Faculty of Medicine Siriraj Hospital, Mahidol University, Bangkok 10700, Thailand; 6 Department of Surgery, Faculty of Medicine Siriraj Hospital, Mahidol University, Bangkok 10700, Thailand

**Keywords:** hepatocellular carcinoma (HCC), alpha-fetoprotein (AFP), small interfering RNA (siRNA), angiogenesis inhibitor, nanoparticle (NP), polylactic-co-glycolic acid (PLGA)

## Abstract

Angiogenesis inhibitor drugs have been explored as important pharmacological agents for cancer therapy, including hepatocellular carcinoma. These agents have several drawbacks, such as drug resistance, nonspecific toxicity, and systemic side effects. Therefore, combination therapy of the drug and small interfering RNA could be a promising option to achieve high therapeutic efficacy while allowing a lower systemic dose. Therefore, we studied adding an alpha-fetoprotein siRNA (*AFP*-siRNA) incorporated on polymeric nanoparticles (NPs) along with angiogenesis inhibitor drugs. The *AFP* siRNA-loaded NPs were successfully synthesized at an average size of 242.00 ± 2.54 nm. Combination treatment of *AFP*-siRNA NPs and a low dose of sunitinib produced a synergistic effect in decreasing cell viability in an in vitro hepatocellular carcinoma (HCC) model. *AFP*-siRNA NPs together with sorafenib or sunitinib greatly inhibited cell proliferation, showing only 39.29 ± 2.72 and 44.04 ± 3.05% cell viability, respectively. Moreover, quantitative reverse transcription PCR (qRT-PCR) demonstrated that *AFP*-siRNA incorporated with NPs could significantly silence *AFP*-mRNA expression compared to unloaded NPs. Interestingly, the expression level of *AFP*-mRNA was further decreased to 28.53 ± 5.10% when sunitinib was added. Therefore, this finding was considered a new promising candidate for HCC treatment in reducing cell proliferation and enhancing therapeutic outcomes.

## 1. Introduction

Liver cancer is the fourth leading cause of cancer-related death worldwide. Among various types of liver cancers, hepatocellular carcinoma (HCC) is a common type of primary liver cancer, causing a major public health problem worldwide [1]. In over 80% of cases, HCC occurs predominantly in patients with underlying chronic liver disease and cirrhosis. The main risk factors of HCC are reported by multifactorial etiology, including extrinsic factors, especially chronic infection with hepatitis B virus (HBV) or hepatitis C virus (HCV), which is the most general risk factor for HCC development, aflatoxin-contaminated food stuff, heavy alcohol intake, obesity, smoking, and type 2 diabetes [2]. Currently, HCC treatments are available in early and intermediate stages and involve surgical resection, liver transplantation, Radio Frequency Ablation (RFA), Microwave Ablation (MWA), and transarterial chemoembolization (TACE). Unfortunately, only 15–20% of HCC patients are suitable for surgical resection or liver transplantation due to the number of lesions and metastases [3,4]. Most patients with HCC are diagnosed at late stages, so treatment with chemotherapy is the only therapeutic option. Several therapeutic drugs, especially antiangiogenesis inhibitors, have advanced development phases for HCC treatment [5]. However, drug resistance, damage to healthy cells, and other side effects such as diarrhea, anorexia, alopecia, abdominal pain, fatigue, and hypertension remained cumbersome. In addition, the difficulty of drug permeability is also a limitation, leading to the poor response of chemotherapeutics treatment [6,7,8].

A significantly high alpha-fetoprotein (AFP) level and a highly vascular tumor are often seen in patients with HCC [9,10]. The AFP may accelerate the proliferation of HCC cells by interacting with the AFP receptor (AFPR), leading to the activation of phosphatidylinositol 3-kinase (PI3K), the protein kinase B in the serine/threonine protein kinases (AKT) and mammalian target of rapamycin (mTOR) pathways [11,12]. This interaction results in the high expression of vascular endothelial growth factor (VEGF), an important mediator in hepatocarcinogenesis by stimulating new blood vessel formation, which leads to HCC invasion and metastasis [2,4,13,14,15,16,17,18]. Monoclonal antibodies against VEGF (i.e., bevacizumab) and intracellular tyrosine kinase inhibitors (TKI) of vascular endothelial growth factor receptors (VEGFR), such as sorafenib, sunitinib, pazopanib, and axitinib, may prevent the activation of the AKT/ERK signaling pathway. They are important pharmacological agents for cancer therapy, including HCC [4,19]. The antiproliferative effect of sunitinib and sorafenib decreases the percentage of HepG2 and HuH7 cell viability. In addition, less proliferation of cancer cells was observed when treated with sunitinib than with sorafenib. Accordingly, sunitinib has become the gold-standard chemotherapeutic drug for HCC, approved by the FDA in 2007 [20]. However, there are direct deleterious effects of antiangiogenesis drugs; for example, bevacizumab, an antiangiogenic drug, could form a complex with VEGF-A and VEGF-R2, leading to an autoactivated VEGF-A–bevacizumab–VEGFR2 complex. This complex could promote cancer cell proliferation, despite the presence of bevacizumab [5,21].

The small interfering ribonucleic acid (siRNA) is a double-stranded RNA with 20–25 nucleotides. The siRNA can bind to the target mRNA and lead to mRNA degradation. It has been widely used in basic research to understand the function of a gene and clinically to inhibit gene expression, curing various human diseases and treating many kinds of cancers such as breast cancer, prostate cancer, and HCC. In HCC research, silencing *AFP* mRNA by siRNA was reported to decrease metastases and abundant apoptotic cells in the HCC cell line [14]. The other studies demonstrated the critical role of cytotoxicity of high *AFP* expression in HCC. Paclitaxel could suppress proliferation in *AFP*-producing cell lines significantly higher than in non-*AFP*-producing cell lines [14,22,23]. Thus, downregulating *AFP* expression using siRNA technology may be the ideal target for HCC therapy and prevention. However, the immune-stimulatory effect, poor intracellular uptake, short biological half-life, and rapid degradation by many enzymes are still limitations for siRNA technology. Therefore, improving strategies for siRNA delivery systems are required for effective HCC treatment. Polylactic-co-glycolic acid (PLGA) nanoparticles has been approved as a drug delivery system in humans by the Food and Drug Administration (FDA) due to its biocompatibility and biodegradability [24] and has demonstrated the potential to deliver nucleic acids in biological systems [25]. Due to its abilities in endosomal escape and sustained release, it has been widely used in siRNA-mediated gene silencing applications [26,27]. Hence, using such nanoparticles as siRNA carriers would mitigate the rapid degradation and poor intracellular internalization effects of siRNA.

In this work, we incorporated the siRNA specific to human *AFP* mRNA (*AFP*-siRNA) into polymeric nanoparticles (NPs) and delivered it to the HepG2 cell line, an *AFP*-producing cell line, to downregulate mRNA expression. Subsequently, the angiogenesis inhibitor drugs were added. The combination treatment of *AFP*-siRNA-incorporated NPs and the angiogenesis inhibitor drugs showed a therapeutic synergistic effect and significantly reduced cell viability, as well as downregulated *AFP* expression compared to the single treatment of either *AFP*-siRNA NPs or angiogenesis inhibitor drugs alone. This study demonstrated a potent strategy for HCC treatment, suggesting that such a combination could be used in humans.

## 2. Results

### 2.1. Effect of Treatment Using AFP-siRNA or/And Angiogenesis Inhibitor Drugs

#### 2.1.1. Antiproliferative Effect of Individual Treatment Using *AFP*-siRNA and/or Angiogenesis Inhibitor Drugs (Sorafenib or Sunitinib)

**Figure 1:**
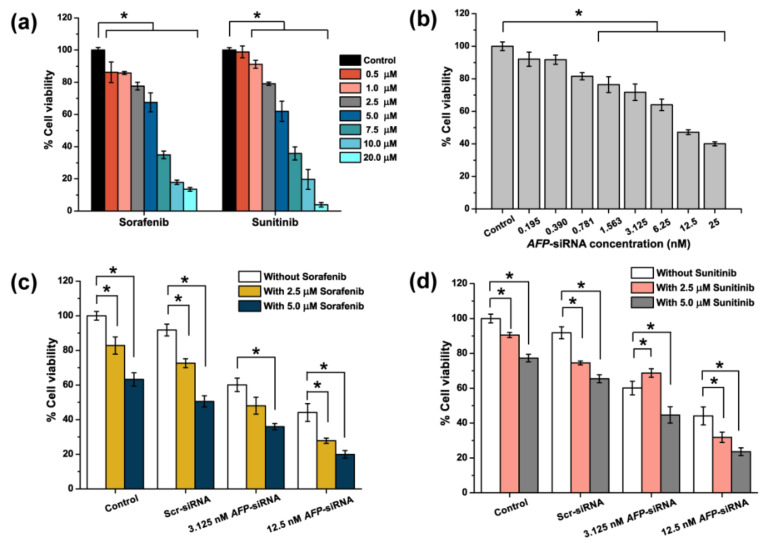
Evaluation of the effect of using antiangiogenetic drugs and/or *AFP*-siRNA on HepG2 cell viability. (**a**) Percentage of cell viability of HepG2 exposed to different concentrations of sorafenib and sunitinib for 24 h. (**b**) Percentage of cell viability of HepG2 exposed to different concentrations of *AFP*-siRNA for 48 h. (**c**) Percentage of cell viability of HepG2 exposed to different concentrations of *AFP*-siRNA followed by sorafenib. (**d**) Percentage of cell viability of HepG2 exposed to different concentrations of *AFP*-siRNA followed by sunitinib. Control (untreated cells) and Scr-siRNA were used as control. Significance was set at *p*-values of *p* < 0.05 and indicated with an asterisk (*).

The antiproliferation potential effect of sorafenib and sunitinib was examined in HepG2 cells at concentrations of 0.50, 1.00, 2.50, 5.00, 7.50, 10.00, and 20.00 μM for 24 h, as shown in Figure 1a. Both drugs exhibited dose-dependent behaviors. The percentage of cell viability was significantly reduced to 86.25 ± 6.38% despite the use of a low dose of sorafenib, i.e., 0.5 μM. Meanwhile, sunitinib began to show a significant effect on HepG2 cells at a concentration of 1.0 μM. When the cells were exposed to 7.5 μM of both drugs, they demonstrated only moderate toxicity in the range of 30–60%. However, increasing the concentration of drugs to 10 μM, severe toxicity was observed with % cell viability of 17.85 ± 1.38 and 19.62 ± 6.16 for sorafenib and sunitinib, respectively. Regarding the acquired data from Figure 1a, sorafenib and sunitinib showed half-maximal inhibitory concentrations (IC50) of 6.02 and 6.15 μM, respectively.

The transfected *AFP*-siRNA using Lipofectamine 2000 at final concentrations of 0.195, 0.390, 0.781, 1.563, 3.125, 6.25, 12.5, and 25 nM for 48 h was performed to evaluate the antiproliferation effect in HepG2 cells. The percentage of cell viability in Figure 1b exhibited a dose-dependent manner of *AFP*-siRNA, i.e., higher concentrations of *AFP*-siRNA were applied, and higher percentages of cell death were observed. The concentration of *AFP*-siRNA at 0.781 nM was nontoxic to the cells, showing 81.56 ± 2.25% of cell viability. However, the cell viability was significantly reduced to 76.41 ± 4.89% when 1.563 nM *AFP*-siRNA was treated and considerably decreased to 40.06 ± 1.26% at the concentration of 25 nM *AFP*-siRNA. According to the result, the IC50 of *AFP*-siRNA was approximately equal to 8.25 nM for the antiproliferation potential effect in the HepG2 cell line.

#### 2.1.2. Cytotoxicity Study of *AFP*-siRNA Combined with Sorafenib or Sunitinib Drugs

The combination effect of sorafenib or sunitinib drugs and *AFP*-siRNA was performed with HepG2 cells through cell viability assays. Scrambled siRNA (Scr-siRNA) and *AFP*-siRNA were transfected into the cells using Lipofectamine 2000. The HepG2 cells were exposed to various concentrations of *AFP*-siRNA starting from 3.125 to 12.5 nM for 48 h prior to the consecutive treatment with angiogenesis inhibitor drugs of 2.50 and 5.0 μM for another 24 h. According to the results in Figure 1c,d, the combination treatment of *AF*P-siRNA and drugs significantly exhibited an enhanced reduction of survival cells compared to the single treatment of either *AFP*-siRNA or drugs. In the case of using sorafenib alone, shown in Figure 1c, the percentages of cell viability of 82.82 ± 4.94% and 63.20 ± 3.81% were observed after treatment with 2.5 and 5.0 μM sorafenib, respectively. Interestingly, treating with 3.125 nM of *AFP*-siRNA, the viability of the cells from combined treatment, *AFP*-siRNA and sorafenib, was reduced by half compared to the treatment of sorafenib alone. The 48.05 ± 4.84% and 35.92 ± 1.82% were attained by using 2.5 and 5.0 μM of sorafenib, respectively. In addition, the cytotoxicity was even improved with a higher concentration treatment of *AFP*-siRNA (12.5 nM). The same effect of combined treatment as sorafenib was observed when sunitinib was exploited, as shown in Figure 1d. The percent cell viability of alone exposure (either *AFP*-siRNA or sunitinib) to the cells was higher than the combined aids of the two therapeutics. When 3.125 nM *AFP*-siRNA was combined with 2.5 or 5.0 μM of sunitinib, the percentage of cell viability was about 68.69 ± 2.40% and 44.65 ± 4.68%, respectively. The toxicity was considerably increased when a higher concentration of *AFP*-siRNA (12.5 nM) was applied, showing only 31.82 ± 2.97% and 23.55 ± 2.23% of viable cells.

### 2.2. Effect of Combination Treatment of siRNA-Loaded Nanoparticles and Angiogenesis Inhibitor Drugs

#### 2.2.1. Characterization of Synthesized Nanoparticles

**Figure 2:**
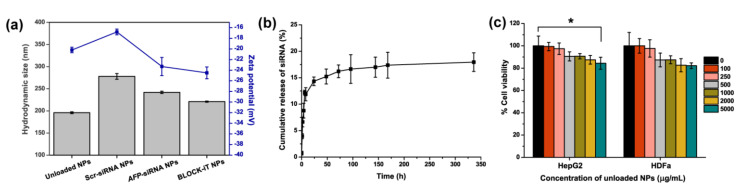
Characteristics of synthesized NPs including unloaded and loaded NPs. (**a**) Hydrodynamic size and zeta potential of unloaded NPs, Scr-siRNA NPs, *AFP*-siRNA NPs, and BLOCK-iT NPs. (**b**) Releasing profile of loaded siRNA over a 14-day period. BLOCK-iT siRNA was used as a representative of *AFP*-siRNA. (**c**) Percentage of cell viability of cancer and normal fibroblast cell lines (i.e., HepG2 and HDFa, respectively) after treatment with different concentrations of unloaded NPs for 24 h. Significance was set at *p*-values of *p* < 0.05 and indicated with an asterisk (*).

Polymeric nanoparticles (NPs) were successfully synthesized using the double emulsion solvent method described in the experimental section. The hydrodynamic size and zeta potential of the NPs were characterized by using Zetasizer. Here, BLOCK-iT^®^, Alexa Fluor^®^ 555 labeled double-stranded RNA (dsRNA) oligomer, was used to represent interested siRNA for evaluation of siRNA encapsulation efficiency and cumulative release studies. Coumarin 6 NPs were also prepared for the determination of the encapsulation efficiency and the cellular uptake study. As shown in Figure 2a, the average hydrodynamic size of the unloaded NPs, Scr-siRNA NPs, *AFP*-siRNA NPs, and BLOCK-iT NPs was about 196.00 ± 2.06, 278.00 ± 6.43, 242.00 ± 2.54, and 221.00 ± 1.32 nm, respectively. The synthesized particles of both unloaded and loaded NPs were negatively charged with the zeta potential of −20.20 ± 0.52, −16.80 ± 0.54, −23.30 ± 1.71, and −24.50 ± 1.11 mV for unloaded NPs, Scr-siRNA NPs, *AFP*-siRNA NPs, and BLOCK-iT NPs, respectively.

The encapsulation efficiency of BLOCK-iT NPs and coumarin 6 NPs were 28.27 ± 3.30% and 50.39 ± 0.62%. Moreover, cumulative release of loaded siRNA was also carried out at 37 °C, as demonstrated in Figure 2b. The result showed an initial burst of 52.29 ± 4.46 % in the first 24 h and a sustained release for 14 days. To verify the suitable concentration of the polymeric NPs as a therapeutic carrier, the cytotoxicity of the various concentrations of unloaded NPs to HepG2 and human dermal fibroblast adult (HDFa) cells was also examined. According to Figure 2c, it can be observed that a high concentration of NPs (up to 5 mg/mL) was treated with both cells for 24 h and still showed a high percentage of viable cells of more than 80% in both HepG2 and HDFa cells.

#### 2.2.2. Cellular Internalization and Cytotoxic Effects of siRNA Nanoparticles on HepG2 Cells

**Figure 3:**
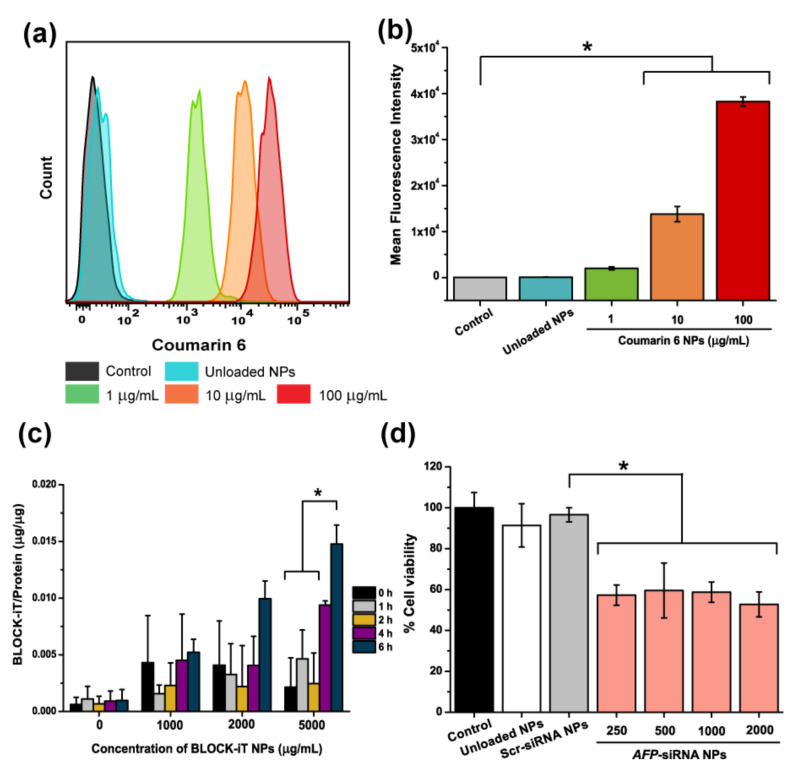
Cellular internalization of siRNA NPs was investigated. (**a**) Histogram analyzed by flow cytometry demonstrated cellular internalized NPs at 1 h. (**b**) Mean Fluorescence Intensity of internalized NPs at different concentrations for 1 h. (**c**) Different concentrations of BLOCK-iT NPs were treated with HepG2 cells at different incubation times. (**d**) The percentage of HepG2 cell viability at different concentrations of *AFP*-siRNA NPs for 48 h. Untreated HepG2 cell, 2000 µg/mL of unloaded NPs, and Scr-siRNA NPs were used as controls. Significance was set at *p*-values of *p* < 0.05 and indicated with an asterisk (*).

In order to verify the ability of NPs as a siRNA carrier, a quantitative analysis from flow cytometry was performed to investigate the cellular internalization. The experiment was carried out at a 1 h incubation time. Firstly, coumarin 6 was loaded into the NPs, obtaining fluorescent NPs which can be tracked by flow cytometer. Different concentrations of coumarin 6 NPs (1, 10, and 100 µg/mL) were incubated with HepG2 for 1 h. Figure 3a demonstrates the successful uptake of coumarin 6-loaded NPs by the cells. Moreover, the result showed a concentration-dependent manner. Moreover, mean fluorescence intensity obtained from flow cytometry in Figure 3b clearly confirmed that when larger content of NPs was used, a higher fluorescence intensity was observed. In addition, BLOCK-iT NPs were picked as a representative of siRNA-loaded NPs. The BLOCK-iT NPs were treated on HepG2 cancer cells to assess the cellular uptake of the siRNA NPs. Figure 3c demonstrated the increase of BLOCK-iT^®^ fluorescence intensities in 1, 2, and 5 mg/mL BLOCK-iT NPs on treated cell line after incubation at different periods.

In addition, the cytotoxic effect of *AFP*-siRNA incorporated with nanoparticles on HepG2 was also assessed. At 48 h of incubation time, the concentrations of 250, 500, 1000, and 2000 μg/mL of *AFP*-siRNA NPs revealed significant cytotoxicity effects, as shown in Figure 3d. The various concentrations of *AFP*-siRNA-loaded NPs showed about 50% cell viability compared with the untreated cells (control). Moreover, all *AFP*-siRNA NPs showed significant difference in cytotoxicity compared to their counterpart one, which is Scr-siRNA NPs. This result confirms that the cytotoxic effect is attributed to *AFP*-siRNA. In contrast, the transfected Scr-siRNA NPs control and empty NPs control showed no toxicity to the cells, even at the high concentration of 2000 μg/mL NPs which was used.

#### 2.2.3. Antiproliferation and Gene Silencing Effects of Sorafenib or Sunitinib in Combination with *AFP*-siRNA Incorporated with Nanoparticles on HepG2 Cells

**Figure 4:**
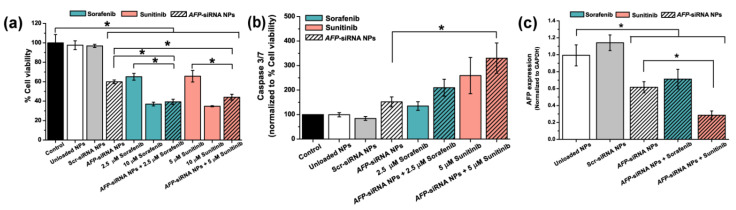
Antiproliferation and gene silencing effects of sorafenib or sunitinib in combination with *AFP*-siRNA loaded NPs on HepG2 cell line. (**a**) Percentage of cell viability of the cells. (**b**) Caspase 3/7 activity of the cells when treated with either single or combined treatments of *AFP*-siRNA NP and drugs. (**c**) Gene silencing effect of combined treatments of siRNA and drugs compared to the single use of *AFP*-siRNA NPs alone. Significance was set at *p*-values of *p* < 0.05 and indicated with an asterisk (*).

The enhanced cytotoxicity of sorafenib or sunitinib combined with *AFP*-siRNA NPs in HepG2 cell is presented in Figure 4a. The cells were transfected with 250 μg/mL *AFP*-siRNA NPs following by the treatment with sorafenib or sunitinib drugs at the synergistic dose. The effect of *AFP*-siRNA NPs combined with 2.5 μM sorafenib revealed about 39.29 ± 2.72% cell viability, while 44.04 ± 3.05% was found when combined with 5.0 μM of sunitinib. Interestingly, the viability of HepG2 cancer cells in all combination treatments was comparable to high dose usage of 10 μM of both sorafenib and sunitinib.

The induction of caspase 3/7 activity was also determined. The result shown in Figure 4b demonstrated caspase 3/7 normalized to the percentage of cell viability. It can be observed that a combination treatment of *AFP*-siRNA NPs and drugs could enhance the caspase 3/7 activity compared to an individual treatment of either siRNA NPs or drug alone. The result demonstrated the significant difference in increasing the caspase 3/7 activity of the combination with *AFP*-siRNA and 5 μM of sunitinib when compared with control, which is *AFP*-siRNA NPs. In addition, the relative *AFP* mRNA expression was also examined using qRT-PCR in HepG2 cells. Figure 4c shows the *AFP* mRNA expression which was normalized to *GAPDH*. Following the result, the treatment of 5.0 μM of sunitinib combined with *AFP*-siRNA NPs dramatically decreased mRNA expression compared to the unloaded NPs. Furthermore, the synergistic treatment could perform better mRNA suppression than the single-treated one, especially for sunitinib. Meanwhile, the silencing effect was not significantly different from the combination 2.5 μM sorafenib and *AFP*-siRNA NPs compared to the single treatment of sorafenib.

## 3. Discussion

This study confirmed that sorafenib and sunitinib, known as angiogenesis inhibitor drugs, significantly suppressed the HepG2 cell viability with IC50 values of 6.02 μM and 6.15 μM (Figure 1a). These results were consistent with a previous study showing the antiproliferation effect on the *AFP*-producing cell line [20]. Despite the powerful therapeutic effect of these drugs, high-dose usage would be required in order to achieve high therapeutic efficacy, which is not clinically useful due to systemic toxicities and drug resistance. Our study suggests that the combination treatment of siRNA therapeutics and angiogenesis inhibitor drugs would be a promising treatment for HCC patients and uses lower, tolerable, dosages of angiogenesis inhibitor drugs. We report designing a siRNA against human *AFP* mRNA that silenced the gene expression in the *AFP*-producing cells, HepG2. Regarding Figure 1b, the result confirmed that the synthesized *AFP*-siRNA is sufficient in enhancing cytotoxicity to the HepG2 cell line with an IC50 value of 8.25 nM. In spite of a low dose of siRNA (1.563 nM), the % cell viability was significantly reduced to 76.41 ± 4.89. Therefore, the obtained result implied an efficiency in using *AFP*-siRNA as a new drug. Our result also indicated that the expression of *AFP* in the studied cell line played an important role in HCC cell survival, consistent with a previous report [23]. Interestingly, the treatment of *AFP*-siRNA in concert with sorafenib or sunitinib significantly reduced the HepG2 cell viability, as illustrated in Figure 1c,d. The synergistic treatment of two therapeutic agents performed better compared to the single treatment of *AFP*-siRNA or drug alone. The interpretation of the synergistic effect was predicted in the condition of 3.125 nM of *AFP*-siRNA combined with 2.5 μM of sorafenib and 5.0 μM of sunitinib. Nevertheless, the 3.125 nM of *AFP*-siRNA with 2.5 μM of sunitinib displayed a slight antagonism. The assumption for these predictions might be caused by the autocrine signaling loop activating the AKT and ERK pathways that promote tumor progression and directly promote VEGF stimulation as the similar mechanism previously described [5,28,29]. Our result successfully presented the synergistic effect of combination treatment between angiogenesis inhibitor and *AFP*-siRNA to antiproliferation in the HCC cell line. However, the combined effect still requires optimization and caution for HCC treatment studies in the future.

However, transfecting siRNA into the cells is difficult, as siRNA cannot penetrate directly into the cells [25]. Therefore, poor cellular uptake, as well as rapid degradation of siRNA, remain challenges for siRNA applications. To overcome the limitation of siRNA delivery to the cells, encapsulated *AFP*-siRNA into polymeric nanoparticles was successfully fabricated. Figure 2a presented the hydrodynamic sizes and zeta potentials of the synthesized particles. Not surprisingly, incorporating siRNA into the nanoparticles made the size of the carrier larger than the unloaded one. Moreover, the high negative charge of the synthetic NPs revealed strong electrostatic repellent interactions between the nanoparticles, indicating the high stability of the delivery system. In addition, the synthesized delivery system also demonstrated a good candidate as a carrier due to its sufficient encapsulation efficiency and suitable drug release profile. Moreover, in Figure 2b, our payload system exhibited a burst release in an initial 24 h, a common biphasic release characteristic of PLGA-based nanoparticles as carriers [30]. Various factors affect the release profile, such as the size, drug–polymer interaction, and molecular weight of the based materials [31]. Weak interaction of negatively charged siRNA and PLGA may cause such a burst phenomenon in the first period. However, the PLGA is still a good candidate as a carrier, as the NPs demonstrated sustained release afterward for the next 14 days. Moreover, the relatively weak interaction with the cells due to repulsion of negative charges possibly caused less toxicity to normal cells [32]. Consistent with our result in Figure 2c, even the high concentration of 5000 μg/mL of unloaded NPs still showed low cytotoxicity to the human dermal fibroblast adult (HDFa). Our finding is also similar to Nurhasni’s work, which demonstrated that empty polymeric nanoparticles showed no toxicity to the fibroblast cells in the concentration range of 0.625–10 mg/mL [33]. These findings made this type of NP a suitable carrier for our *AFP*-siRNA. 

To confirm the ability of the NPs to deliver siRNA into the cells, cellular internalization was quantitatively analyzed by flow cytometry. Regarding Figure 3a,b, the results expressed the successful cellular uptake, with 100% engulfment at the 1 h incubation time, indicating a potent candidate as a carrier. Not surprisingly, it was also found that when higher number of fluorescent-tagged NPs was applied, the higher fluorescence intensity was acquired. However, our particles have shown the great preference by cells, as they could be taken up despite a short time applied. Meanwhile, only 59% of siRNA-loaded PLGA NPs from previous report could be accumulated [34]. In addition, the amount of taken BLOCK-iT/protein was also performed to confirm the internalization of the particles. Not surprisingly, Figure 3c illustrated that the particles could be taken up by the cells at the time-dependent and dose-dependent manners. The moderate percentage of transfection efficiency found in this system may be attributed to the repulsion of the negative charges between the particles and the cell membranes. In addition, the great efficiency for the antiproliferation effect in HepG2 cells, up to 50% was presented in Figure 3d. These results also confirmed that PLGA NPs can deliver *AFP*-siRNA to HepG2 cells and inhibit cell growth by downregulating the PI3K/AKT signal pathway, thereby contributing to slowing down cell proliferation, as described in the studies by Xiaoping et al. and Zhu et al [22,35]. All of the above results ensured the PLGA NPs were safe and showed high potential strategy to deliver *AFP*-siRNA for *AFP*-producing cells. Nevertheless, achieving higher encapsulation efficiency and cellular uptake can be improved for future studies by modifying the PLGA nanoparticles with the cationic poly-L-lysine (PLL) or polyethyleneimine (PEI), or other manners that are described in other studies [34,36].

Interestingly, this is the first report that *AFP*-siRNA-incorporated PLGA nanoparticles possessed a synergistic effect with angiogenesis inhibitor drug to inhibit the viability of HepG2 cancer cells without apoptotic cascade activation. Our study, in Figure 4a, demonstrated that this combination treatment dramatically decreased the HepG2 cell viability even though a low dose of the angiogenesis drug was used. The cell viability of the combined treatment was doubly decreased compared to the single treatment of each drug. Instead of using a high-dose drug (10 µM of each drug), the combined effect of siRNA and low-dose drug (2.5 µM and 5.0 µM for sorafenib and sunitinib, respectively) performed comparable antiproliferation effects to the single treatment of either *AFP*-siRNA or drugs, confirming an improvement of the therapeutic effects by reducing the dosage. These actions were reported in Zhu et al., that the combination treatment of paclitaxel and *AFP*-siRNA can decrease HLE cell growth and increase the apoptosis caspase 3 cascade [22]. Interestingly, the induction of cell death was not monitored in *AFP*-siRNA encapsulated in PLGA NPs when combined with sorafenib or sunitinib (Figure 4b). Herein, increasing evidence that the delivery system using PLGA NPs is safe for healthy cells by reducing toxicity, not inducing cell apoptosis. In addition, the PLGA NPs can enhance the ability to deliver *AFP*-siRNA to HCC cells and are highly efficient in reducing HCC cell growth in our study. The potential effect of the combination treatment was confirmed by observing the *AFP* mRNA expression level in HepG2 cells, as shown in Figure 4c. Remarkably, *AFP*-siRNA NPs combined with sunitinib showed a significantly downregulating *AFP* mRNA expression. However, combined with sorafenib, it can only silence mRNA expression levels in the same way as a single treatment of *AFP*-siRNA NPs or the drug alone.

## 4. Materials and Methods

### 4.1. Materials

The chemical synthesis of siRNA, 2′Fluoro modified at base C, and U (2′F) was purchased from GenePharma (Shanghai, China). Lipofectamine^®^ 2000 transfection reagent, TRIzol, BLOCK-iT^TM^ Fluorescent Oligo, and SuperScript^®^ III First-Strand Synthesis System were purchased from Invitrogen (Carlsbad, CA, USA). PLGA, lactide/glycolide (50:50), MW: 38,000–54,000, and PVA, MW: 30,000–70,000 were purchased from Sigma (St. Louis, MO, USA). The HepG2 cell line was purchased from the Cell Lines Service (Eppelheim, Germany). The HDFa cell line was bought from American Type Culture Collection (ATCC, Manassas, VA, USA). Modified Eagle Medium (DMEM), Fetal bovine serum (FBS), and penicillin–streptomycin (Pen-Strep) were received from Gibco (Grand Island, NY, USA). Sorafenib and sunitinib were obtained from Abcam (Cambridge, MA, USA). 

### 4.2. Preparation of Chemical Synthesis of Small Interfering RNA (siRNA) 

The siRNA sequences against to human *AFP* gene (*AFP*-siRNA) with scrambled (Scr-siRNA) as negative control were designed, and the sequences of the synthesized oligonucleotides are: *AFP*-sense, 5′GCCACUUACAAGGAAGUAAGCAA3′, *AFP*-antisense, 5′GCUUAC-UUCCUUGUAAGUGGCUU3′, Scr-sense,5′GCAGGGUGGCGACCA CGUCUU3′, and Scr-antisense, 5′GACGUGGUCGCCACCCUGCUU3′. The sense and antisense strands of siRNA were annealed by mixing in an equal volume in an annealing buffer, followed by heating at 95 °C for 2 min and cooling down slowly at room temperature for 20 min to generate a double-stranded siRNA.

### 4.3. Cell Cultures

The HepG2 and HDFa were cultured in Dulbecco’s Modified Eagle Medium (DMEM) supplemented with 10% Fetal bovine serum (FBS), 100 U/mL penicillin, and 100 µg/mL streptomycin at 37 °C in 5% CO_2_ incubator.

### 4.4. In Vitro Cytotoxicity Study of AFP-siRNA, Angiogenesis Inhibitor Drugs, and Combined Treatment of AFP-siRNA and Drugs 

The transfected *AFP*-siRNA into HepG2 cells was performed at the concentrations of 0.195, 0.390, 0.781, 1.563, 3.125, 6.25, 12.5, and 25 nM using the Lipofectamine^®^ 2000 transfection reagent according to the manufacturer’s instructions. After incubating for 48 h, the in vitro cytotoxicity was determined using CellTiter-Blue^®^ cell viability assay following the the manufacturer’s protocol (Promega, Madison, WI, USA).

The angiogenesis inhibitor drugs, sorafenib or sunitinib, were added to HepG2 cells at the concentrations of 0.5, 1.0, 2.5, 5.0, 7.5, 10.0, and 20.0 μM, incubated for 24 h. The in vitro cytotoxicity of each condition was examined by cell viability assay.

To evaluate the effect of combined therapeutics, HepG2 cells were firstly transfected by *AFP*-siRNA at concentrations of 3.125 and 12.5 nM using the Lipofectamine^®^ 2000 for 48 h according to the manufacturer’s protocol. Then, sorafenib or sunitinib at 2.50 and 5.0 μM were treated and consecutively incubated for another 24 h. The cell viability assay was performed to examine the cell toxicity. Finally, the combined effect of *AFP*-siRNA and antiangiogenesis drugs were quantified.

### 4.5. Synthesis of siRNA Loaded Nanoparticles 

The siRNA-loaded nanoparticles (siRNA NPs) were prepared using the double emulsion solvent evaporation method. Briefly, the primary emulsion (w/o) was prepared by adding siRNA with annealing buffer into 1.5 mL of chloroform solution containing PLGA and emulsified for 30 s by using ultrasonic probe sonicate (Sonics and Materials Inc., Newtown, CT, USA) with 30% of amplitude in an ice bath. Next, the mixture was emulsified in 5% *w/v* PVA solution in RNase-free water, then sonicated with 70% of amplitude for 5 min, and then stirred overnight. The siRNA NPs were recovered by using Sorvall RC6+ centrifuge (ThermoFisher Scientific, Asheville, NC, USA) at 15,000 rpm for 30 min, and the formulated nanoparticles were washed and lyophilized. Finally, the nanoparticles loaded with siRNA were obtained. BLOCK-iT^®^ Fluorescent Oligo (Alexa Flour^®^ 555) and coumarin 6 were loaded in NPs using a similar procedure.

### 4.6. Characterization of siRNA Loaded Nanoparticles

Hydrodynamic size and zeta potential of the nanoparticles were measured using the zetasizer Nano ZS (Malvern Instruments Ltd., Malvern, Worcestershire, UK). Each measurement was performed in triplicate at 25°C. The indirect encapsulation efficiency of siRNA-loaded NPs was determined using BLOCK-iT^®^-loaded NPs and quantified at Ex555/Em595 using Multi-Detection Microplate Readers (Bio-Tek Instrument Inc., Winooski, VT, USA). The drug release studies of siRNA were performed using BLOCK-iT^®^-encapsulated NPs at 37 °C. At each time point, samples were taken and calculated the released fraction of BLOCK-iT^®^ at Ex555/Em595 by using Multi-Detection Microplate Readers (Bio-Tek Instrument Inc., Winooski, VT, USA).

### 4.7. In Vitro Cytotoxicity Study of Nanoparticles 

HepG2 and HDFa cells were incubated with unloaded NPs at various concentrations for 24 h. The cell viability assay was performed to determine the toxicity of nanoparticles to the cells. In addition, *AFP*-siRNA NPs were transfected to HepG2 cells for 48 h to examine the HepG2 cell viability. 

### 4.8. Intracellular Uptake and Accumulation of siRNA-PLGA Nanoparticles in HepG2 

Coumarin 6 nanoparticles at concentrations of 1, 10, and 100 µg/mL were incubated with the cells for 1 h. The cellular uptake of coumarin 6-loaded NPs at the interested period was quantitatively confirmed using BD FACSCalibur (San Jose, CA, USA).

In addition, BLOCK-iT^®^ nanoparticles at concentrations of 1000, 2000, and 5000 µg/mL were added to the cells and incubated for 1, 2, 4, and 6 h. The amount of internalized nanoparticles were detected by using Multi-Detection Microplate Readers at Ex555/Em595. 

### 4.9. Combination Analysis of Antiangiogenesis Drug with AFP-siRNA NPs

HepG2 cells were treated with *AFP*-siRNA NPs at a concentration of 250 µg/mL for 48 h. To determine the effect of the combination treatments of antiangiogenesis drugs with *AFP*-siRNA NPs, the cells were treated with sorafenib or sunitinib and further incubated for 24 h, followed by measuring CellTiter-Blue^®^ Cell Viability Assay and caspase 3/7 activity using Caspase-Glo^®^ 3/7 reagent (Promega, Madison, WI, USA) according to the manufacturer’s protocols. 

### 4.10. RNA extraction and quantitative Reverse Transcription Polymerase Chain Reaction (qRT-PCR) 

Total RNA was extracted using TRIzol^®^ reagent according to the manufacturer’s instructions. Reverse transcription was performed using the SuperScript^®^ III First-Strand Synthesis System from Invitrogen under the condition described in the manufacturer’s instructions. A quantitative PCR reaction was performed on the Stratagene Mx3005P real-time thermal cyclers using KAPA SYBR^®^ FAST qPCR Master Mix (2X) Kit from Roche (Wilmington, MA, USA) with the following conditions: 95 °C for 10 min followed by 40 cycles of 94 °C for 30 s, 57 °C for 30 s, and 72 °C for 30 s. The relative mRNA expression was analyzed according to the 2^−△△Ct^ method. Following primers were used: *AFP* forward primer: 5′CCTTCCTGTATGCACCTACAAT3′, *AFP* reverse primer: 5′AACTGTTG-CTGCCTTTGTTTG3′, *GAPDH* forward primer: 5′AGCCACATCGCTCAGACAC3′, *GAPDH* reverse primer: 5′GTTAAAAGCAGCCTTGGTGA3′. 

### 4.11. Statistical Analysis

Data are presented as mean ± S.D. of three independent experiments if not specified. One-way ANOVA statistical analysis with post-hoc comparisons test was performed by SPSS Statistics 18.0. Significance was set at *p*-values of *p* < 0.05 and indicated with an asterisk (*). 

## 5. Conclusions

At a low dose of sorafenib, sunitinib, and minimum concentration of *AFP*-siRNA, the combination of *AFP*-siRNA-encapsulated PLGA nanoparticles and angiogenesis inhibitors have a synergistic effect on silencing *AFP* expression and antiproliferation, leading to a significant decrease in cell survival on HepG2 cancer cells without the induction of caspase 3/7 activity. Therefore, our study overcame the limitations of the high-dose usage of angiogenesis-inhibitory drugs by lowering its toxicity with the aid of siRNA-loaded NPs. Accordingly, using polymeric nanoparticles loaded with *AFP*-siRNA and combined with sunitinib could be a promising alternative strategy to enhance the potential of HCC treatment by alleviating the toxicity and therapeutic drug dose. Moreover, this would contribute to cost-effectiveness for cancer treatment and prolong the life expectancy of patients. 

## Data Availability

The data presented in this study are available within the article.

## References

[1] Bray F., Ferlay J., Soerjomataram I., Siegel R.L., Torre L.A., Jemal A. (2018). Global cancer statistics 2018: GLOBOCAN estimates of incidence and mortality worldwide for 36 cancers in 185 countries. CA Cancer J. Clin..

[2] Dhanasekaran R., Bandoh S., Roberts L.R. (2016). Molecular pathogenesis of hepatocellular carcinoma and impact of therapeutic advances. F1000Research.

[3] Chang A.Y., Wang M. (2013). In-vitro growth inhibition of chemotherapy and molecular targeted agents in hepatocellular carcinoma. Anti-Cancer Drugs.

[4] Llovet J.M., Villanueva A., Lachenmayer A., Finn R.S. (2015). Advances in targeted therapies for hepatocellular carcinoma in the genomic era. Nat. Rev. Clin. Oncol..

[5] Simon T., Gagliano T., Giamas G. (2017). Direct Effects of Anti-Angiogenic Therapies on Tumor Cells: VEGF Signaling. Trends Mol. Med..

[6] Neul C., Schaeffeler E., Sparreboom A., Laufer S., Schwab M., Nies A.T. (2016). Impact of Membrane Drug Transporters on Resistance to Small-Molecule Tyrosine Kinase Inhibitors. Trends Pharmacol. Sci..

[7] Pascale F., Bedouet L., Baylatry M., Namur J., Laurent A. (2015). Comparative Chemosensitivity of VX2 and HCC Cell Lines to Drugs Used in TACE. Anticancer Res..

[8] Fu Y., Wei X., Lin L., Xu W., Liang J. (2018). Adverse reactions of sorafenib, sunitinib, and imatinib in treating digestive system tumors. Thorac. Cancer.

[9] Chen T., Dai X., Dai J., Ding C., Zhang Z., Lin Z., Hu J., Lu M., Wang Z., Qi Y. (2020). AFP promotes HCC progression by suppressing the HuR-mediated Fas/FADD apoptotic pathway. Cell Death Dis..

[10] Liu K., Zhang X., Xu W., Chen J., Yu J., Gamble J.R., McCaughan G.W. (2017). Targeting the vasculature in hepatocellular carcinoma treatment: Starving versus normalizing blood supply. Clin. Transl. Gastroenterol..

[11] Zheng L., Gong W., Liang P., Huang X., You N., Han K.Q., Li Y.M., Li J. (2014). Effects of AFP-activated PI3K/Akt signaling pathway on cell proliferation of liver cancer. Tumor Biol..

[12] Karar J., Maity A. (2011). PI3K/AKT/mTOR Pathway in Angiogenesis. Front. Mol. Neurosci..

[13] Kim K.I., Chung H.K., Park J.H., Lee Y.J., Kang J.H. (2016). Alpha-fetoprotein-targeted reporter gene expression imaging in hepatocellular carcinoma. World J. Gastroenterol..

[14] Lu Y., Zhu M., Li W., Lin B., Dong X., Chen Y., Xie X., Guo J., Li M. (2016). Alpha fetoprotein plays a critical role in promoting metastasis of hepatocellular carcinoma cells. J. Cell Mol. Med..

[15] Wang S., Zhu M., Wang Q., Hou Y., Li L., Weng H., Zhao Y., Chen D., Ding H., Guo J. (2018). Alpha-fetoprotein inhibits autophagy to promote malignant behaviour in hepatocellular carcinoma cells by activating PI3K/AKT/mTOR signalling. Cell Death Dis..

[16] Li M., Li H., Li C., Wang S., Jiang W., Liu Z., Zhou S., Liu X., McNutt M.A., Li G. (2011). Alpha-fetoprotein: A new member of intracellular signal molecules in regulation of the PI3K/AKT signaling in human hepatoma cell lines. Int. J. Cancer.

[17] Takahashi M., Sato T., Sagawa T., Lu Y., Sato Y., Iyama S., Yamada Y., Fukaura J., Takahashi S., Miyanishi K. (2002). E1B-55K-deleted adenovirus expressing E1A-13S by AFP-enhancer/promoter is capable of highly specific replication in AFP-producing hepatocellular carcinoma and eradication of established tumor. Mol. Ther..

[18] Avci M.E., Konu O., Yagci T. (2008). Quantification of SLIT-ROBO transcripts in hepatocellular carcinoma reveals two groups of genes with coordinate expression. BMC Cancer.

[19] Boucher E., Forner A., Reig M., Bruix J. (2009). New drugs for the treatment of hepatocellular carcinoma. Liver Int..

[20] Ho H.K., Chau B.T., Wong W., Lim K.S., Teo V., Ong H.T., Chen X., Zhang W., Hui K.M., Go M.L. (2014). Benzylidene-indolinones are effective as multi-targeted kinase inhibitor therapeutics against hepatocellular carcinoma. Mol. Oncol..

[21] Alesini D., Mosillo C., Naso G., Cortesi E., Lacovelli R. (2015). Clinical experience with everolimus in the second-line treatment of advanced renal cell carcinoma. Ther. Adv. Urol..

[22] Zhu M., Li W., Lu Y., Dong X., Chen Y., Lin B., Xie X., Guo J., Li M. (2016). Alpha fetoprotein antagonizes apoptosis induced by paclitaxel in hepatoma cells in vitro. Sci. Rep..

[23] Meng W., Li X., Bai Z., Yuan J., Liu T., Yan J., Zhou W., Zhu K., Zhang H., Li Y. (2014). Silencing alpha-fetoprotein inhibits VEGF and MMP-2/9 production in human hepatocellular carcinoma cell. PLoS ONE.

[24] Cun D., Jensen D.K., Maltesen M.J., Bunker M., Whiteside P., Scurr D., Foged C., Nielsen H.M. (2011). High loading efficiency and sustained release of siRNA encapsulated in PLGA nanoparticles: Quality by design optimization and characterization. Eur. J. Pharm. Biopharm..

[25] Chernikov I.V., Vlassov V.V., Chernolovskaya E.L. (2019). Current Development of siRNA Bioconjugates: From Research to the Clinic. Front. Pharmacol..

[26] Patil Y., Panyam J. (2009). Polymeric nanoparticles for siRNA delivery and gene silencing. Int. J. Pharm..

[27] Raichur A., Nakajima Y., Nagaoka Y., Matsumoto K. (2015). Strategist PLGA Nano-capsules to Deliver siRNA for Inhibition of Carcinoma and Neuroblastoma Cell Lines by Knockdown of MYC Proto-oncogene using CPPs and PNA. NanoWorld J..

[28] Peng S., Wang Y., Peng H., Chen D., Shen S., Peng B., Chen M., Lencioni R., Kuang M. (2014). Autocrine vascular endothelial growth factor signaling promotes cell proliferation and modulates sorafenib treatment efficacy in hepatocellular carcinoma. Hepatology.

[29] Fragoso R., Elias A.P., Dias S. (2007). Autocrine VEGF loops, signaling pathways, and acute leukemia regulation. Leuk. Lymphoma.

[30] Chigumira W., Maposa P., Gadaga L.L., Dube A., Tagwireyi D., Maponga C.C. (2015). Preparation and Evaluation of Pralidoxime-Loaded PLGA Nanoparticles as Potential Carriers of the Drug across the Blood Brain Barrier. J. Nanomater..

[31] Yoo J., Won Y.Y. (2020). Phenomenology of the Initial Burst Release of Drugs from PLGA Microparticles. ACS Biomater. Sci. Eng..

[32] Shao X.R., Wei X.Q., Song X., Hao L.Y., Cai X.X., Zhang Z.R., Peng Q., Lin Y.F. (2015). Independent effect of polymeric nanoparticle zeta potential/surface charge, on their cytotoxicity and affinity to cells. Cell Prolif..

[33] Nurhasni H., Cao J., Choi M., Kim I., Lee B.L., Jung Y., Yoo J.W. (2015). Nitric oxide-releasing poly(lactic-co-glycolic acid)-polyethylenimine nanoparticles for prolonged nitric oxide release, antibacterial efficacy, and in vivo wound healing activity. Int. J. Nanomed..

[34] Risnayanti C., Jang Y.S., Lee J., Ahn H. (2018). PLGA nanoparticles co-delivering MDR1 and BCL2 siRNA for overcoming resistance of paclitaxel and cisplatin in recurrent or advanced ovarian cancer. Sci. Rep..

[35] Wang X., Wang Q. (2018). Alpha-Fetoprotein and Hepatocellular Carcinoma Immunity. Can. J. Gastroenterol. Hepatol..

[36] Cai C., Xie Y., Wu L., Chen X., Liu H., Zhou Y., Zou H., Liu D., Zhao Y., Kong X. (2017). PLGA-based dual targeted nanoparticles enhance miRNA transfection efficiency in hepatic carcinoma. Sci. Rep..

